# The Effect of Binaural Beat Audio on Operative Pain and Anxiety in Cataract Surgery under Topical Anaesthesia: A Randomized Controlled Trial

**DOI:** 10.3390/ijerph191610194

**Published:** 2022-08-17

**Authors:** Ling Jiunn Loong, Koh Koon Ling, Evelyn Li Min Tai, Yee Cheng Kueh, Garry Kuan, Adil Hussein

**Affiliations:** 1 Department of Ophthalmology, School of Medical Sciences, Universiti Sains Malaysia, Kubang Kerian 16150, Kelantan, Malaysia; 2 Biostatistics and Research Methodology Unit, School of Medical Sciences, Health Campus, Universiti Sains Malaysia, Kubang Kerian 16150, Kelantan, Malaysia; 3 Exercise and Sports Science, School of Health Sciences, Universiti Sains Malaysia, Kubang Kerian 16150, Kelantan, Malaysia

**Keywords:** binaural beats, cataract surgery, phacoemulsification, topical anaesthesia, pain, anxiety

## Abstract

Background: The aim of this paper was to examine the analgesic and anxiolytic effects of binaural beat audio in patients undergoing cataract surgery under topical anaesthesia. Methods: This was a prospective, randomized controlled trial of 61 patients undergoing cataract surgery under topical anaesthesia. They were divided into two research conditions; the binaural beat audio group, and a sham-control grou*p* (ear phones with no music). Patients completed the State-Trait Anxiety Inventory questionnaire (STAI), and their blood pressure (BP) and heart rate (HR) were measured pre- and post-intervention. Intraoperative pain levels were ascertained using a visual analog scale (VAS) completed immediately after the surgery. Results: The binaural beat grou*p* had significantly lower pain scores (*p* < 0.001), HR (*p* < 0.001), diastolic BP (*p* = 0.003), mean arterial pressure (*p* = 0.007) and anxiety (*p* = 0.009) than the control group. Within the binaural beat group, subjects experienced a statistically significant reduction in HR (*p* = 0.004) and anxiety (*p* < 0.001) levels compared to baseline values, while all parameters, except anxiety, increased significantly in the control group. Conclusions: Binaural beat audio decreases operative pain and anxiety in cataract surgery under topical anaesthesia. It may have additional benefits in modulating the tachycardic response to stress.

## 1. Introduction

Topical anaesthesia is the preferred choice of anaesthesia among patients undergoing phacoemulsification cataract surgery [1]. Although it avoids the inherent complications of needle-based local anaesthesia, such as chemosis and subconjunctival haemorrhage [1,2], there is evidence that it is less effective for pain relief than those techniques, including retrobulbar [3], peribulbar [1,3] and sub-Tenon anaesthesia [3,4,5,6,7]. Addition of intracameral anaesthesia reduces pain perception compared to topical anaesthesia alone, but intra-operative pain levels are still higher than with Sub-Tenon anaesthesia [8,9]. Furthermore, topical anaesthesia does not address intra-operative anxiety, which may exacerbate the subjective experience of pain [9,10]. Intravenous sedation with midazolam has, likewise, been found to have no significant effect on reduction of pain or anxiety [11]. Thus, there remains an unmet need for adjunctive therapies to reduce anxiety and pain during phacoemulsification under topical anaesthesia.

Sound has complex effects on the brain, with both audible and inaudible frequencies exerting changes on electroencephalogram-documented brainwaves [12,13,14,15]. Brainwave entrainment in the form of auditory binaural beats has been shown to have analgesic [16,17,18] and anxiolytic effects [19,20,21]. Binaural beats are an auditory processing artefact created by the interference between two similar frequencies of sound presented to each ear via earphones [22,23]. Disparate audible tones of up to 1000 hertz (Hz) frequency, differing by less than approximately 30 Hz, are processed by the brain via binaural integration, allowing perception of a third, subliminal tone [24,25]. The frequency of this illusory tone is equal to the mean frequency of the two stimuli, while its amplitude fluctuates with a frequency amounting to the difference between the stimuli [26]. For example, playing a 400 Hz tone in the right ear and a 410 Hz tone in the left will entrain the brain towards a beat frequency of 10 Hz, which is in the alpha range associated with relaxation [27].

The analgesic effects of binaural beats have been observed in acute and chronic pain. For example, Zampi et al. demonstrated that patients with chronic pain who listened to binaural beats 20 min a day for a fortnight experienced significant reductions in perceived pain [18]. In addition, two randomized trials evaluating the use of binaural beats for intra-operative nociception control found that patients under general anaesthesia required significantly less intraoperative fentanyl when listening to binaural beats than when listening to non-binaural beat music or a blank tape [16,17]. In addition, Dabu-Bondoc et al. also observed significantly lower post-operative pain scores in the binaural beat group than in the other groups [16]. Similarly, binaural beat music has been demonstrated to have superior anxiolytic effects not only over blank headphones, but over music without binaural beats [21]. A meta-analysis of binaural beats in pain perception and anxiety concluded that these beats have a medium, significant, consistent effect size in reducing anxiety levels and pain perception [19].

Binaural beats embedded in music have been observed to decrease patient-reported, and physiological measures of, anxiety in patients undergoing phacoemulsification under local anaesthesia [25]. However, as the aforementioned study was performed under retrobulbar anaesthesia, the efficacy of binaural beat audio for pain management has not been explored. Our study thus aimed to evaluate the analgesic and anxiolytic effects of binaural beat audio in patients undergoing cataract surgery under topical anaesthesia.

## 2. Materials and Methods

We conducted a prospective, randomized controlled trial in Hospital Universiti Sains Malaysia from April 2017 to March 2018. The study was approved by the Human Research Ethics Committee of Universiti Sains, Malaysia (USM/JEPeM/16090291). The conduct of the study followed the tenets of the declaration of Helsinki.

### 2.1. Participants

The study recruited 62 patients. Data was collected by a single researcher. Inclusion criteria were subjects with senile cataract undergoing phacoemulsification with intraocular lens implantation under topical anaesthesia. Exclusion criteria included previous intraocular surgery, pre-existing concomitant ocular disease (e.g., glaucoma), hearing impairment, neurological disease, psychiatric disease, use of anxiolytic medications, and presence of intraoperative complications necessitating conversion to extracapsular cataract extraction.

Patients were randomized into two groups using the random sampling envelope technique; binaural beats with topical anaesthesia (intervention group) and topical anaesthesia alone (sham-control group, wearing earphones with no audio). Patients were blinded to allocation until administration of interventions. During the procedure, one patient from the control group was excluded because of intraoperative intraocular complications resulting in conversion of phacoemulsification to extra capsular cataract extraction under sub-tenon block.

### 2.2. Sample Size Calculation

In the present study, the sample size calculation was conducted using G*Power 3.1.9.7 (Heinrich Heine University, Dusseldorf, Germany) based on the analysis of variance (ANOVA): repeated measures between factors (i.e., intervention and control groups; group effect), within factors (time effect), and within–between factors (interaction effect). The largest sample size was described in this section. The parameters used for the sample size calculation were effect size = 0.20, alpha value = 0.05, statistical power = 0.80, number of groups = 2, number of measurements = 2 times. The estimated sample size was 31 per group with total sample size of 62.

### 2.3. Subjective Assessment of Pain

Pain was assessed immediately after the surgery using a visual analog scale (VAS). The VAS is a reliable and widely used method of measuring intraoperative pain in cataract surgery [26,27,28,29]. It consists of a linear scale with its ends corresponding to the extreme limits of the measured response [30,31,32].

### 2.4. Subjective Assessment of Anxiety

Subjects completed the 6-item State-Trait Anxiety Inventory (STAI) before and after the surgery [33,34]. The STAI is a valid, sensitive and reliable measure of anxiety in cataract surgery, with higher scores reflecting higher anxiety levels. [27,35,36,37,38]. The questionnaire presents equal numbers of anxiety-present (e.g., ‘tense’, ‘worried’) and anxiety-absent (e.g., ‘calm’, ‘relaxed’) items to each patient, requiring patients to answer each question with one the following responses: (1) Never, (2) Some, (3) Much or (4) Completely.

### 2.5. Objective Assessment of Pain and Anxiety

Blood pressure (BP) and heart rate (HR) were recorded at the start and at the end of surgery (Spacelabs Ultraview SL Monitoring System, Spacelabs Healthcare, Issaquah, Washington). Although pain and anxiety are subjective experiences, BP and HR have been correlated with pain and anxiety during cataract surgery and may act as proxy measures [25,39].

### 2.6. Interventions

**Figure 1:**
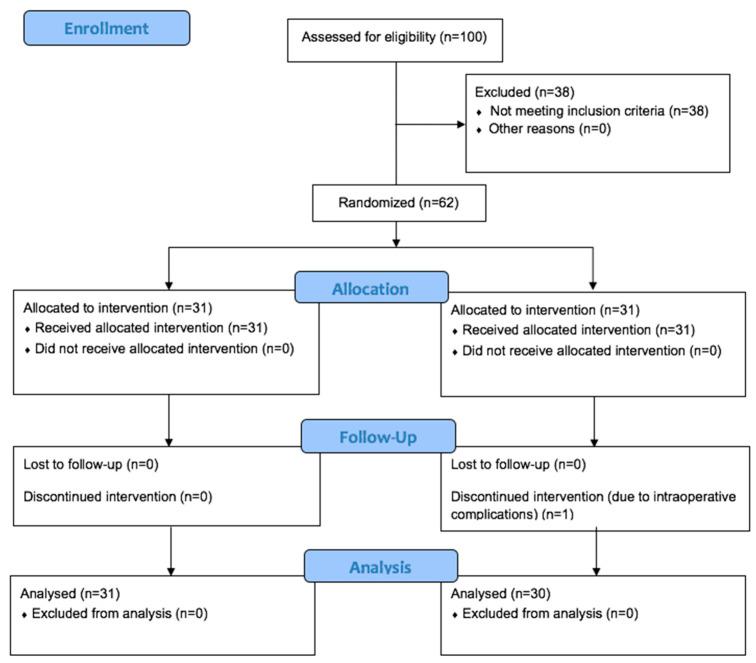
Consort Diagram on participants’ allocation.

After answering the STAI, patients were randomly assigned to two groups, both provided with an identical MP3 player and canal-type stereo earphones (see Figure 1). In the intervention group, binaural beats (Happiness Frequency 10 Hz Binaural Beats, Greenred Production) were utilized, while the control group had no audio. Earphones were positioned 10 min before the start of surgery.

The dilation regime was topical tropicamide 1% (Mydriacyl, Alcon, Puurs, Belgium) and phenylephrine hydrochloride 2.5% (Mydfrin, Alcon, Fort Worth, TX, USA). Topical anaesthesia consisted of proparacaine hydrochloride 0.5% (Alcaine, Alcon, Puurs, Belgium). All patients were also given non-preserved intracameral lidocaine hydrochloride 1% at the commencement of the surgery. No oral or intravenous sedation was used. Phacoemulsification was performed in the standard manner by a single surgeon blinded to allocation group. Immediately after the surgery, patients completed the VAS, followed by the STAI.

### 2.7. Statistical Analysis

Statistical Package for the Social Sciences (SPSS) version 27.0 (IBM Corp., Armonk, NY, USA) was used for the data analysis. The data consisted of the two groups (i.e., binaural beat audio vs. control) with a two-time measurement of the outcome variables (i.e., systolic BP, diastolic BP, mean arterial pressure, HR, STAI score). Independent sample *t*-test and Pearson chi-square statistic were used to compare numerical and categorical variables between groups. Paired sample *t*-test was used for pre- and post-test measurements within each group. Mixed factorial analysis of variance (ANOVA) was conducted to examine the effects of the intervention. The effect examined included the time, group, and interaction (time × group) effects. The ‘time’ effect evaluated the difference between pre-operative and post-operative outcome variables within each group, while the ‘group’ effect compared the differences in outcome variables between groups. The ‘time × group’ effect allowed comparison of the changes in outcome variables among the groups over time. A *p*-value of <0.05 was taken as a significant result.

## 3. Results

A total of 61 patients were included in the study; 31 in the binaural beat group and 30 in the control group (Figure 1). Baseline clinical and socio-demographic factors were comparable between the groups, as were the STAI scores (Table 1 and Table 2). There was no significant difference in surgical duration between the groups (*p* = 0.879).

The binaural beat group had significantly lower pain scores (mean = 1.52, SD = 0.93) than the control group (mean = 2.60, SD = 1.19, *p* < 0.001). The HR, DBP, MAP and anxiety scores were also significantly lower in the former group than in the latter (Table 2). Pain score was significantly correlated with SBP, DBP, MAP and anxiety score within the control group, but not in the intervention group (Table 3).

**Figure 2:**
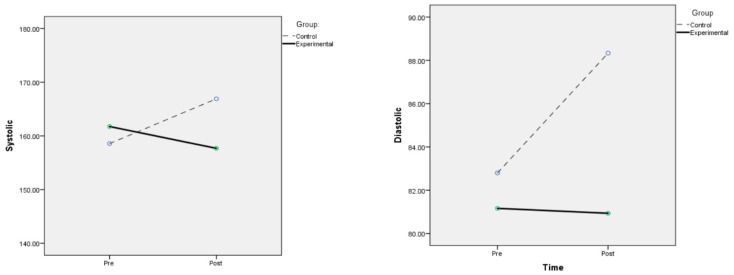

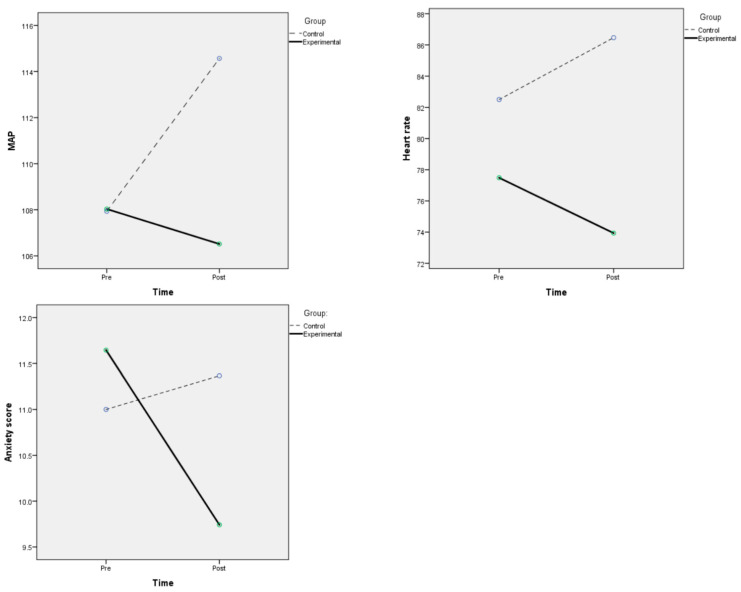
Mean values of outcomes variables for control and experimental groups at pre- and post-test.

There were significant differences in all outcome measures within each group over time (Table 4). The binaural beat group experienced reductions in SBP, DBP, MAP, HR and anxiety, whereas the control group suffered elevations in these parameters (Figure 2). Paired t test showed that these changes were statistically significant for all variables in the control group, with the exception of anxiety (SBP, *p* < 0.001; DBP, *p* < 0.001; MAP, *p* < 0.001; HR, *p* < 0.001; anxiety score, *p* = 0.190; see Table 5). In the binaural beat group, the decremental trend of all outcome variables from pre- to post-test was statistically significant only for heart rate and anxiety (SBP, *p* = 0.133; DBP, *p* = 0.862; MAP, *p* = 0.362; HR, *p* = 0.004; anxiety score, *p* < 0.001; see Table 5).

## 4. Discussion

Cataract surgery is unique among other surgeries for the ease and safety with which it can be performed under topical anesthesia, thus avoiding the risks of regional blocks [5,40,41,42]. Unfortunately, intraoperative pain control is still suboptimal compared to sub-tenon anaesthesia, even with the addition of intracameral injection [3,4,5,6,7,43], necessitating an on-going search for further modalities to enhance patient comfort during cataract surgery under topical anaesthesia. Binaural beat-embedded music has previously been shown to reduce systolic BP, HR and anxiety levels in patients undergoing cataract surgery under retrobulbar block [25]. Our study builds on this foundation by demonstrating that binaural beat audio alone not only has anxiolytic effects and physiologic, but also reduces pain perception among patients undergoing cataract surgery under topical anaesthesia.

Our intervention group had significantly lower pain scores than the control group, reflecting reduced intraoperative pain in those exposed to binaural beat audio. Binaural beats have previously been demonstrated to reduce perceived pain during surgery under general anaesthesia [16,17,44]. To the best of our knowledge, this is the first study to document the efficacy of binaural beats as analgesic adjuncts to topical anaesthesia in sedation-free cataract surgery.

Preoperative anxiety levels have been found to predict pain during cataract surgery [45]. Pain-relevant anxiety amplifies pain perception by modulating the response of the entorhinal cortex of the hippocampal formation [10]. Our study observed that binaural beat audio had analgesic effects which may potentially be independent of its anxiolytic effects. In line with the literature, our binaural beat group had significantly lower post-intervention anxiety scores than the control group. Binaural beat audio has positive effects on anxiety, whether used regularly [46,47] or as a one-off intervention [20,21]. Its effect on decreasing operative anxiety among patients undergoing phacoemulsification has also been recognized [25]. Our study concurs with the results of that study, in which the binaural beat group had significantly reduced BP and HR compared to the control group [25].

BP and HR act as physiological indicators of anxiety [48,49,50] and pain [27,39,51]. These parameters, as well as the post intervention-STAI scores, were found to increase in the control group and decrease in the binaural beat group. The lack of correlation between pain scores and these physiologic measures within the binaural group were in contrast to the correlations observed among these parameters in the control group. Binaural beats may reduce BP and HR by increasing parasympathetic dominance [52,53]. This may have particular utility in cataract surgery, in which tachycardia is a risk factor for complications, such as suprachoroidal haemorrhage.

Binaural beats are auditory brainstem responses generated from the superior olivary nucleus in response to different frequency auditory stimuli provided to each ear [54]. A recent meta-analysis revealed that they exert effects on cognition, memory and psychophysiological states like anxiety [55]. Anxiety levels have been correlated with vestibulo--cortical hemispheric dominance [56]. By integrating the contralateral auditory system, binaural beats may thus mediate their effects via synchronization of the brain hemispheres [22,57] and alter functional connectivity [58]. Binaural beats may also affect the autonomic nervous system, increasing parasympathetic activation and facilitating sympathetic withdrawal [53].

Strengths of our study were its use of validated instruments to measure pain and anxiety, allowing direct comparison with other published studies in this field. Furthermore, by performing surgery under topical anaesthesia, our hemodynamic parameters were free from the fluctuations which may occur with peribulbar injection or sedation [59]. The main limitation of our study was that our control group could only be masked until the administration of the intervention, resulting in possible bias. Besides that, our control group of earphones without audio may have experienced anxiolytic effects due to reduction in ambient sound, thus underestimating the beneficial effect of binaural beat audio in routine cataract surgery. In addition, the effect of binaural beats on nociception may appear amplified due to our lack of a ‘music-only’ control group; music has also been demonstrated to reduce intra and postoperative pain, although not to as great an extent as binaural beat audio [16,17]. Finally, we acknowledge that the effect size of our intervention ranged from small to medium (i.e., 0.01 to 0.27), indicating that the differences between groups were modest. Further studies evaluating supplemental methods of analgesia in cataract surgery under topical anaesthesia are warranted.

Topical anaesthesia is rapidly becoming the standard of care for daycare cataract surgery [2,44]. Although intravenous sedation has been successfully used as an adjunct to these cases, it entails increased cost and resources, which are not readily available at many centers [28,60,61]. Patient cooperation is essential for surgery under topical anaesthesia, and can be affected by pain and anxiety [29,61]. We demonstrated that binaural beat audio has the potential to decrease operative pain, anxiety, BP and HR. Further studies should evaluate the cost-effectiveness of this intervention as an adjunct to cataract surgery under topical anaesthesia.

## 5. Conclusions

Binaural beat audio has analgesic and anxiolytic effects in patients undergoing cataract surgery under topical anaesthesia. Its additional parasympathetic effects may be particularly beneficial in cataract surgery.

## Figures and Tables

**Table 1:** Comparison of baseline socio-demographic variables between groups.

Category	Overall *n* (%)	Control (*n* = 30)		Intervention (*n* =31)		*p*-Value
		*n* (%)	Mean (SD)	*n* (%)	Mean (SD)	
Age	65.8 (7.9)		63.9 (6.2)		67.7 (9.0)	0.069 **
Gender						0.15 *
Male	33 (54.1)	19 (63.3)		14 (45.2)		
Female	28 (45.9)	11 (36.7)		17 (54.8)		

* analyzed by Pearson Chi-Square, *p* < 0.05 significant. ** analyzed by Independent Sample *t*-test, *p* < 0.05 significant.

**Table 2:** Mean difference of study variables between control and experimental groups based on time.

Study Variables	Time	Mean Difference (95% *CI*)	*p*-Value
Systolic BP	Baseline/pre	−3.18 (−11.58, 5.23)	0.453
	Post	9.22 (−0.71, 19.16)	0.068
Diastolic BP	Baseline/pre	1.64 (−3.27, 6.55)	0.507
	Post	7.40 (2.71, 12.09)	0.003
MAP	Baseline/pre	−0.10 (−5.31, 5.12)	0.970
	Post	8.05 (2.28, 13.82)	0.007
Heart rate	Baseline/pre	5.02 (−1.41, 11.44)	0.124
	Post	12.53 (7.05, 18.02)	<0.001
Anxiety	Baseline/pre	−0.65 (−1.79, 0.50)	0.265
	Post	1.63 (0.42, 2.84)	0.009

*CI* = Confidence interval; *p*-value based on Independent Sample *t*-test.

**Table 3:** Correlation between pain score and outcome measures.

Pain Score	Post- Systolic	Post-Diastolic	Post-MAP	Post- Heart Rate	Post-Anxiety
Control	*r* = 0.44	*r* = 0.40	*r* = 0.51	*r* = 0.35	*r* = 0.47
	*p* = 0.015	*p* = 0.029	*p* = 0.004	*p* = 0.055	*p* = 0.008
Experimental	*r* = −0.15	*r* = 0.02	*r* = −0.08	*r* = 0.11	*r* = 0.25
	*p* = 0.415	*p* = 0.928	*p* = 0.666	*p* = 0.542	*p* = 0.170

*r* = Pearson correlation value, *p* = *p*-value.

**Table 4:** Descriptive statistics and effects of intervention in control and experimental groups over time.

Variables	Group	Pretest	Posttest	Time			Group			Time*Group		
		Mean (SD)	Mean (SD)	*F*	*p-*Value	*η*2	*F*	*p-*Value	*η*2	*F*	*p-*Value	*η*2
Systolic	Con	158.57 (11.31)	166.90 (13.14)	1.71	0.196	0.03	0.50	0.485	0.01	14.41	<0.001	0.19
	Exp	161.74 (20.14)	157.68 (23.92)									
Diastolic	Con	82.80 (6.41)	88.33 (7.25)	8.36	0.005	0.12	4.16	0.046	0.07	9.84	0.003	0.14
	Exp	81.16 (11.85)	80.94 (10.66)									
MAP	Con	107.93 (6.07)	114.57 (7.54)	5.95	0.018	0.09	2.44	0.123	0.04	15.09	<0.001	0.20
	Exp	108.03 (12.96)	106.52 (13.93)									
Heart rate	Con	82.50 (11.19)	86.47 (9.13)	0.06	0.811	0.01	9.44	0.003	0.14	18.62	<0.001	0.24
	Exp	77.48 (13.71)	73.94 (12.03)									
Anxiety	Con	11.00 (2.42)	11.37 (2.11)	10.23	0.002	0.15	0.83	0.366	0.01	22.32	0.001	0.27
	Exp	11.65 (2.04)	9.74 (2.58)									

Con = control, Exp = Experimental, *η*2 = effect size; *F* = F statistic; Mixed Factorial ANOVA was used.

**Table 5:** Mean differences between pretest and posttest within control and experimental groups.

Variables	Group	Mean Difference Pretest-Posttest	95% *CI*	*p*-Value
Systolic	Con	−8.33	−12.23, −4.44	<0.001
	Exp	4.06	−1.31, 9.44	0.133
Diastolic	Con	5.53	−8.20, −2.86	<0.001
	Exp	0.23	−2.41, 2.86	0.862
MAP	Con	−6.63	−9.29, −3.97	<0.001
	Exp	1.52	−1.83, 4.86	0.362
Heart rate	Con	−3.97	−6.70, −1.23	<0.001
	Exp	3.55	1.25, 5.84	0.004
Anxiety	Con	−0.37	0.93, −1.34	0.190
	Exp	1.90	1.10, 2.70	<0.001

Paired Sample *t*-test was used.

## Data Availability

The data is available upon request from the authors.
